# Curcumin Inhibits Mitochondrial Injury and Apoptosis from the Early Stage in EAE Mice

**DOI:** 10.1155/2014/728751

**Published:** 2014-04-27

**Authors:** Jinzhou Feng, Tao Tao, Weiping Yan, Cindy Si Chen, Xinyue Qin

**Affiliations:** ^1^Department of Neurology, The First Affiliated Hospital of Chongqing Medical University, Chongqing 400016, China; ^2^Department of Neurology, Affiliated Hospital of Luzhou Medical College, Luzhou 646000, China; ^3^Department of Medicine, Drexel University College of Medicine, Philadelphia, PA 19129, USA

## Abstract

The exact pathophysiological change concerning mitochondrial injury and oligodendrocyte apoptosis in MS and EAE model is still unknown. Whether curcumin is able to inhibit mitochondrial injury and suppress the apoptosis in the early stages of MS/EAE is still unclear. We first explored mitochondrial injury and apoptosis at different time points p.i. in C57 BL/6 EAE mice. We then explored the effects of curcumin on mitochondria and apoptosis. Results showed that mitochondrial injury can be observed 3 days p.i. Apoptosis in the spinal cord occurred 3 days p.i. and the apoptotic cells were shown to be oligodendrocytes and neuronal cells. Curcumin significantly reduced the number of apoptotic cells and inhibited the upregulation of cyt-c, caspase-9, and caspase-3 at 7 days p.i. in the EAE mice. These observations demonstrate that mitochondrial injury and oligodendrocyte/neuronal apoptosis occur in the early stages of EAE. Curcumin can inhibit apoptosis in EAE mice which maybe act through protection of mitochondrial injury and inhibition of the intrinsic apoptotic pathway.

## 1. Introduction

The fundamental pathophysiology of newly formed lesions in multiple sclerosis (MS) is still not very clear, though it is generally attributed to demyelination events caused by the T-cell-mediated immune response [1]. Recently, studies on autopsy specimen found that the initial pathological findings with the formation of new lesions are different from the traditional MS pathological findings. Oligodendrocyte apoptosis is the earliest pathological change in new lesions [2–4]. However, the mechanism for oligodendrocyte apoptosis is still unknown. The experimental autoimmune encephalomyelitis (EAE) model is a classical animal model for MS, but the relationship between apoptosis and the typical pathological changes in the EAE model is still unclear. Mitochondrial injury has been identified as an important nonimmune mechanism in the pathology of MS [5]. Mitochondrial dysfunction and a higher demand of energy have been found in MS [6, 7]. Whether mitochondrial injury is involved in the early pathology of MS/EAE is still unknown.

Curcumin is a polyphenolic phytochemical isolated from the rhizome of the plant* Curcuma longa* which naturally is not present in the normal brain. It has been shown to have a neuroprotective role in EAE [8, 9], but it is unknown whether curcumin is able to protect mitochondria from injury and subsequently suppress apoptosis in the early pathology of MS/EAE.

In this study, we explored apoptosis and mitochondrial injury in C57 BL/6 EAE mice at different time points postimmunization (p.i.). We also explored the mechanism by which curcumin inhibits apoptosis, which occurs through the protection of mitochondrial injury during the early stages of EAE. This study aimed to elucidate the early pathological events of MS/EAE and to find an effective intervention for the early stages of MS.

## 2. Materials and Methods

### 2.1. Reagents and Antibodies

Bacillus calmette-guerin (BCG) and pertussis were purchased from the National Institutes for Food and Drug Control of China. MOG33-35 peptide was purchased from CL. Bio-scientific Company. Primary antibodies to CNPase, MAP-2, MBP, Cyt-c, cleaved caspase-3, and cleaved caspase-9 were purchased from Abcam. TUNEL in suit apoptosis detection kit was purchased from Roche.

### 2.2. Animals

6–8-week-old female C57BL/6 mice were obtained from the Experimental Animal Center of Chongqing Medical University. All experiments followed the guidelines of the International Council for Laboratory Animal Science. This study was approved by the Ethics Committee of Chongqing Medical University, Chongqing, China. All groups were randomly assigned and all assessments were double blinded to investigators.

### 2.3. Induction and Clinical Evaluation of EAE

To induce EAE, mice were subcutaneously immunized with a 0.2 mL emulsion consisting of 300 *μ*g/mL of MOG 33-55 peptide and an equal volume of complete Freund's adjuvant (CFA). The mice were also intraperitoneally injected with 500 ng of pertussis toxins on days 0 and 2 p.i. All procedures were conducted under anesthesia with 3.5% chloral hydrate. The clinical score was evaluated each day using the standard 0–5 scale [10, 11]; 0: unaffected, 1: tail limpness, 2: failure to right on attempt to roll over, 3: partial paralysis, 4: complete paralysis, and 5: moribund.

### 2.4. Groups and Treatments

To access the early pathological events of EAE, mice were divided into normal, 3-day, 7-day, 16-day (acute stage), and 32-day (chronic stage) p.i. groups. No other special treatments were given to any of the 4 experimental groups after EAE was induced.

In order to explore the role of curcumin, mice were divided into normal, EAE, control, and curcumin groups. The last 3 groups were divided into 3-day, 7-day, 16-day, and 32-day groups p.i. The mice received i.p. injection of curcumin at 200 mg/kg/d dissolved in 0.5% methylcellulose (curcumin group) or 0.5% methylcellulose (control group) when they were immunized [12].

### 2.5. TUNEL Assay

To quantify apoptosis, T24 xenograft sections were processed for in situ immunocytochemical localization of nuclei exhibiting DNA fragmentation using the technique of terminal deoxynucleotidyl transferase- (TdT-) mediated dUTP digoxigenin nick-end labeling (TUNEL) in conjunction with an apoptosis detection kit (In Situ Cell Death Detection Kit, POD). The protocols were followed according to the manufacturer's instructions.

Paraffin-embedded samples (3 *μ*m thick sections) were deparaffinized and rehydrated with xylene and ethanol and permeabilized with 20 µg/mL of proteinase K for 30 min. Endogenous peroxidase was inactivated by coating the samples with 3% H_2_O_2_. Sections were rinsed with PBS and then immersed for 60 min in TdT buffer at 37°C and then incubated for 30 min with the antidigoxigenin peroxidase conjugate, followed by the peroxidase substrate (3′-diaminobenzidine tetrahydrochloride [DAB]). Finally, sections were counterstained with 0.5% (wt/vol) methyl green. The apoptosis index was evaluated using a light microscope. Results were expressed as a percentage of TUNEL-positive cells to the total cells [13].

### 2.6. Transmission Electron Microscope Examination (TEM)

All biopsy specimens were fixed in glutaraldehyde solution followed by 1% osmium tetroxide and then embedded in resin block. Semithin 500 nm sections were cut by the ultramicrotome and stained with toluidine blue. After light microscopic orientation, 70 nm ultrathin sections were cut and stained by uranyl acetate and lead citrate [14]. The ultrathin sections were then examined by a transmission electron microscope (Hitachi-7500).

### 2.7. Immunofluorescence Double Labeling

Paraffin-embedded sections were deparaffinized, rehydrated in a graded series of ethanol, and then incubated in H_2_O_2_ (0.3%, 15 min). For antigen retrieval, sections were treated with 10 mmol/L sodium citrate buffer (pH 6.0) and heated with a microwave oven for 20 min. Tissue was permeabilized with 0.5% Triton X-100, and then sections were incubated in 5% goat serum for 30 min at room temperature. Sections were then incubated with MAP2 (1 : 500) or CNPase (1 : 1000) primary antibodies at 4°C overnight. Next, sections were washed and incubated with tetramethylrhodamine isothiocyanate- (TRITC-) conjugated goat anti-mouse IgG (1 : 100) in the dark for 60 min, and then DAPI was added for 5 min. After washing with PBS, TUNEL mixture solution was added [15]. The results were observed with a fluorescence microscope (Lecia, Germany). TUNEL-positive cells were labeled with green fluorescence, and MAP2 (+) or CNPase (+) cells were labeled with red fluorescence. Images were processed with Image-Pro 6.0 software.

### 2.8. Western Blot

Western blot was performed using antibodies that specifically recognized MBP(1 : 1000)/cleaved caspase-3(1 : 5000)/cleaved caspase-9(1 : 500)/cyt-c (1 : 500). Equal amounts of sample protein were loaded for SDS-PAGE/immunoblot analysis. Mouse monoclonal antibody for *β*-actin (1 : 2000) was used as a loading control. Relative protein expression levels were reflected by the band density of target proteins relative to *β*-actin [16].

### 2.9. Statistical Analysis

All results were presented as means ± standard deviation (SD). Statistical differences between groups were compared using one-way analysis of variance (ANOVA). Statistical analysis was performed using SPSS 18.0. A *P* value < 0.05 was considered statistically significant.

## 3. Results

### 3.1. Mitochondrial Injury Occurred in the Early Stages after EAE Was Immunized

**Figure 1:**
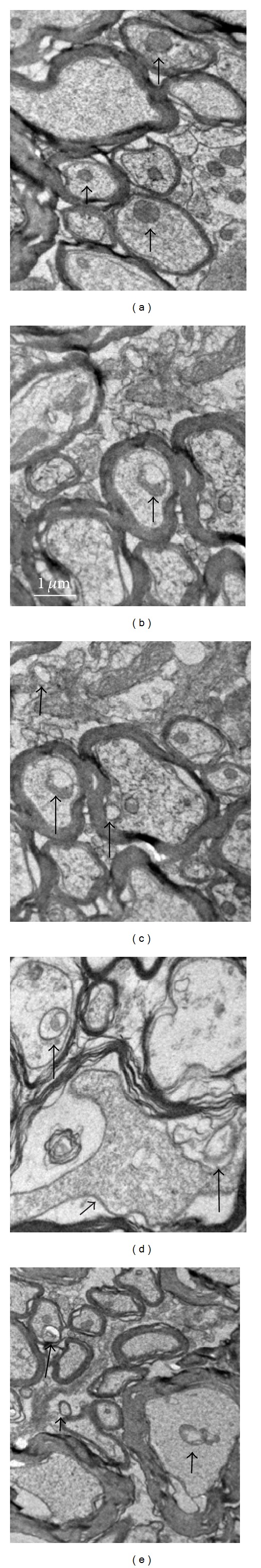
Mitochondrial injury in the spinal cord p.i. was detected by EM. (a) Normal group, (b) 3-day group, (c) 7-day group, (d) 16-day group, and (e) 30-day group. Arrows indicate mitochondrial injury ((a)–(e)) and axoplasmic atrophy (d); scale bar = 1 *μ*m. The morphology of mitochondria, myelin sheath, and axons was normal in the normal group (a). Swollen mitochondria and compressed crista can be observed with normal myelin sheath and axons in the 3-day and 7-day groups ((b)-(c)). Mitochondria are shown with swelling, reduction of mitochondrial crest, and matrix density or vacuolation in the 16-day group. Demyelination and axoplasmic atrophy can also be observed (d). Mild alleviation of lesions is observed in the 30-day group (e) compared to the 16-day group.

To explore the early pathological changes after EAE was immunized, the morphological characteristics of mitochondria, myelin sheath, and axons in the spinal cord were observed by TEM. Both mitochondrial crista structure and membranes were clear when observed in the normal group. The morphology of mitochondria, myelin sheath, and axons was normal. At 3 days p.i, swollen mitochondria and compressed crista were seen, while the structure of the myelin sheath and axons was still normal. At 16 days p.i, the pathological changes were more severe. Mitochondria were swollen and there was either reduction of mitochondrial crest and matrix density or vacuolation. Demyelination and axoplasmic atrophy were also observed. The lesions in the 30-day group were similar, with mild alleviation compared to the 16-day group (Figure 1).

### 3.2. Cell Apoptosis Occurred in the Early Stages after EAE Was Immunized

**Figure 2:**
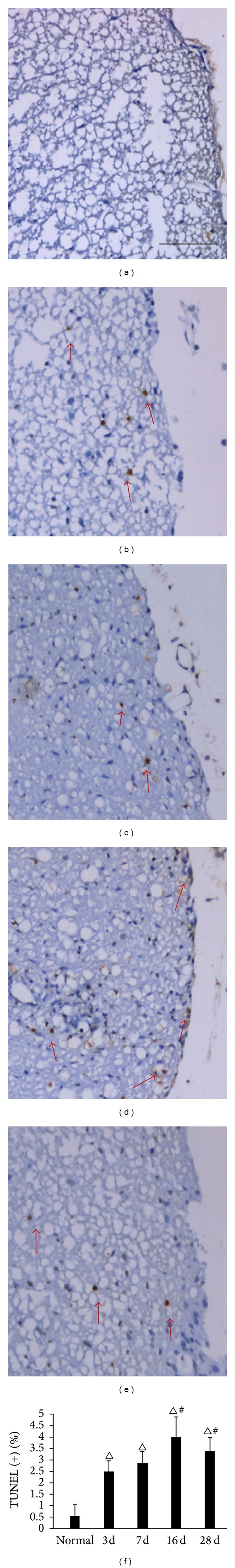
Expression of TUNEL (+) apoptosis cells in the spinal cord of EAE mice at different time points. (a) Normal group, (b) 3-day group, (c) 7-day group, (d) 16-day group, (e) 30-day group, and (f) Quantitative analysis of the number of TUNEL (+)/total cells (*n* = 6). Arrows indicate TUNEL (+) cells; ^△^ *P* < 0.01, compared to the normal group; ^#^ *P* < 0.05, compared to the 3-day group. Scale bar = 200 *μ*m.

Analysis of apoptotic cells at different time points post-EAE was performed using TUNEL. No obviously TUNEL (+) cells were observed in the normal group. After EAE was immunized, TUNEL (+) cells were observed as early as 3 days p.i. However, no significant differences were observed in the number of TUNEL (+) cells between the 3-day and 7-day groups (*P* > 0.05). The TUNEL (+) cells reached maximum levels at 16 days p.i. (*P* < 0.05) and largely expressed around the blood vessels. The expression level in the 30-day group was lower than in the 16-day group (*P* > 0.05) (Figure 2 and Table 1).

### 3.3. Localization of Apoptotic Cells in the Early Stages after EAE Was Immunized

**Figure 3:**
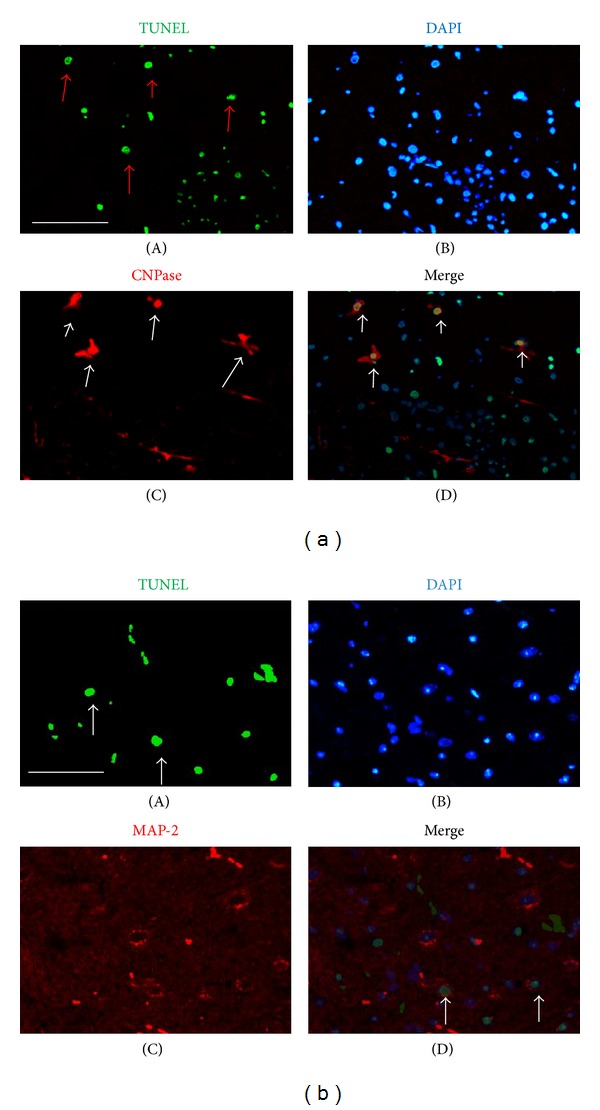
(a) Colocalization with an anti-oligodendrocytes antibody (CNPase) and TUNEL (+) cells by double-labeled immunofluorescence in the spinal cord 3 days p.i. (A) TUNEL (+) cells (green), (B) DAPI stain showing the nucleus (blue), (C) anti-oligodendrocyte antibody (CNPase) (red), and (D) merged image of (A)–(C). Arrows indicate TUNEL (+) cells (A), CNPase (+) cells (C), or the colocalized cells (D). Scale bar = 100 *μ*m. (b) Colocalization with anti-neuron antibody (MAP-2) and TUNEL (+) cells by double-labeled immunofluorescence in the spinal cord 3 days p.i. (A) TUNEL (+) cells (green), (B) DAPI stain showing the nucleus (blue), (C) anti-MAP-2 antibody (red), and (D) merged image of (A)–(C). Arrows indicate TUNEL (+) cells (A), MAP-2 (+) cells (C), or the colocalized cells (D). Scale bar = 100 *μ*m.

We localized the TUNEL-positive cells with CNPase/MAP-2-positive cells (a specific marker of neural/oligodendroglia) via immunofluorescence double labeling. The results showed that CNPase/MAP-2 (+) cells were colocalized with TUNEL (+) cell in the spinal cord 3 days after EAE was immunized. The results suggested that the apoptotic cells in the early stages post-EAE were oligodendroglia and neurons (Figure 3).

### 3.4. Curcumin Inhibits Cell Apoptosis in EAE

**Figure 4:**
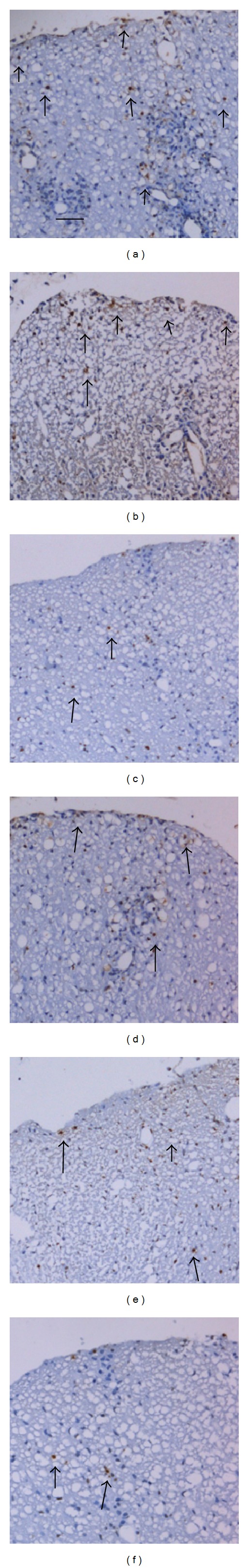
Expression of TUNEL (+) apoptosis cells in the spinal cord of EAE mice in different groups. (a) EAE group (16d), (b) control group (16d), (c) curcumin group (16d), (d) EAE group (30d), (e) control group (30d), and (f) curcumin group (30d); arrows indicate TUNEL (+) cells. Scale bar = 100 *μ*m.

The effect of curcumin on apoptosis in the EAE model was evaluated by TUNEL. The results showed that, in both the acute stage (16 days) and chronic stage (30 days) of EAE, the number of TUNEL (+) cell in the curcumin group was significantly lower than the EAE group and control group at the same time points (Figure 4 and Table 2).

### 3.5. MBP Expression Level Detected by Western Blot

**Figure 5:**
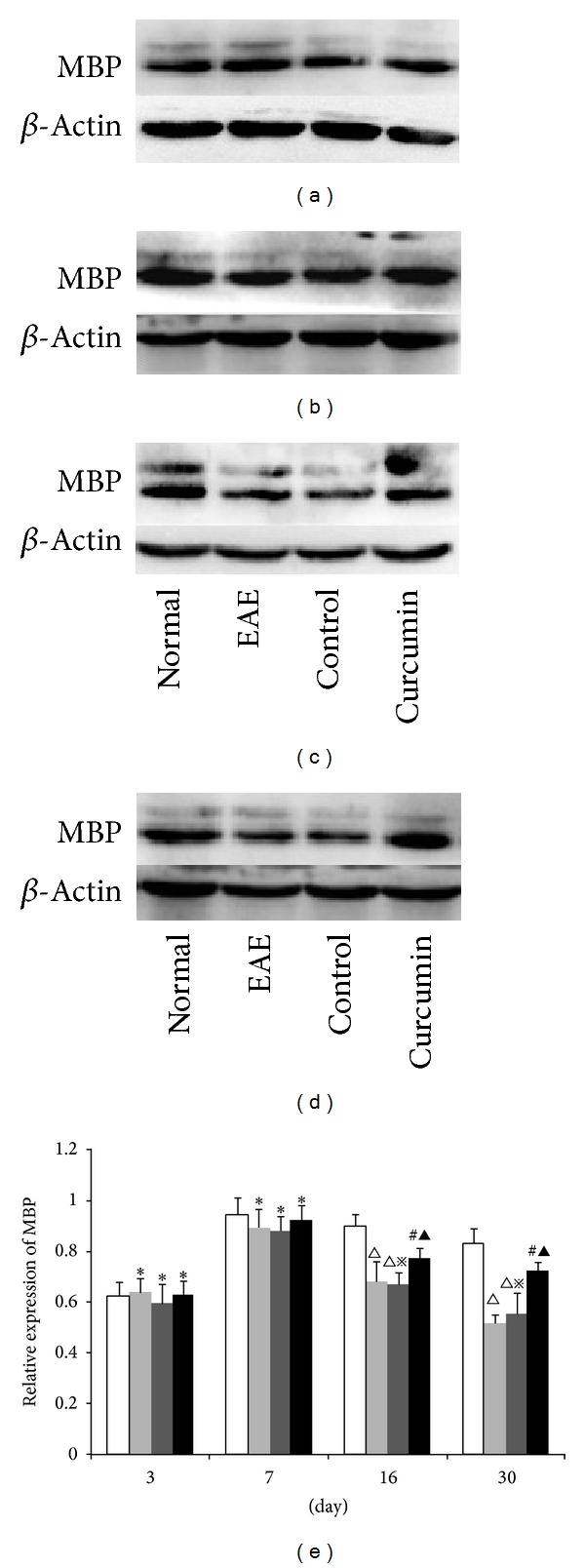
Expression of MBP in each group detected by western blot. (a) 3-day group, (b) 7-day group, (c) 16-day group, and (d) 30-day group. (e) Bars represent relative protein expression of MBP related to *β*-actin. Data are presented as mean ± SD. *P > 0.05, compared to the normal group; ^△^ *P* < 0.01, compared to the normal group; ^#^ *P* < 0.05, compared to the normal group; ^▲^ *P* < 0.05, compared to the EAE group at the same time point; ^*※*^ *P* > 0.05, compared to the EAE group at the same time point.

The MBP protein expression level in the spinal cord was evaluated by western blot. No significant differences were observed at 3 days or 7 days between the normal and the EAE groups (*P* > 0.05). The expression of MBP in the EAE group was significantly lower than the normal group at the 16-day and 30-day time points (*P* < 0.05). The expression of MBP in the curcumin group was significantly higher than in the EAE group at the same time points (*P* < 0.05) (Figure 5).

### 3.6. Cleaved-Caspase-3 Expression Levels Detected by Western Blot

**Figure 6:**
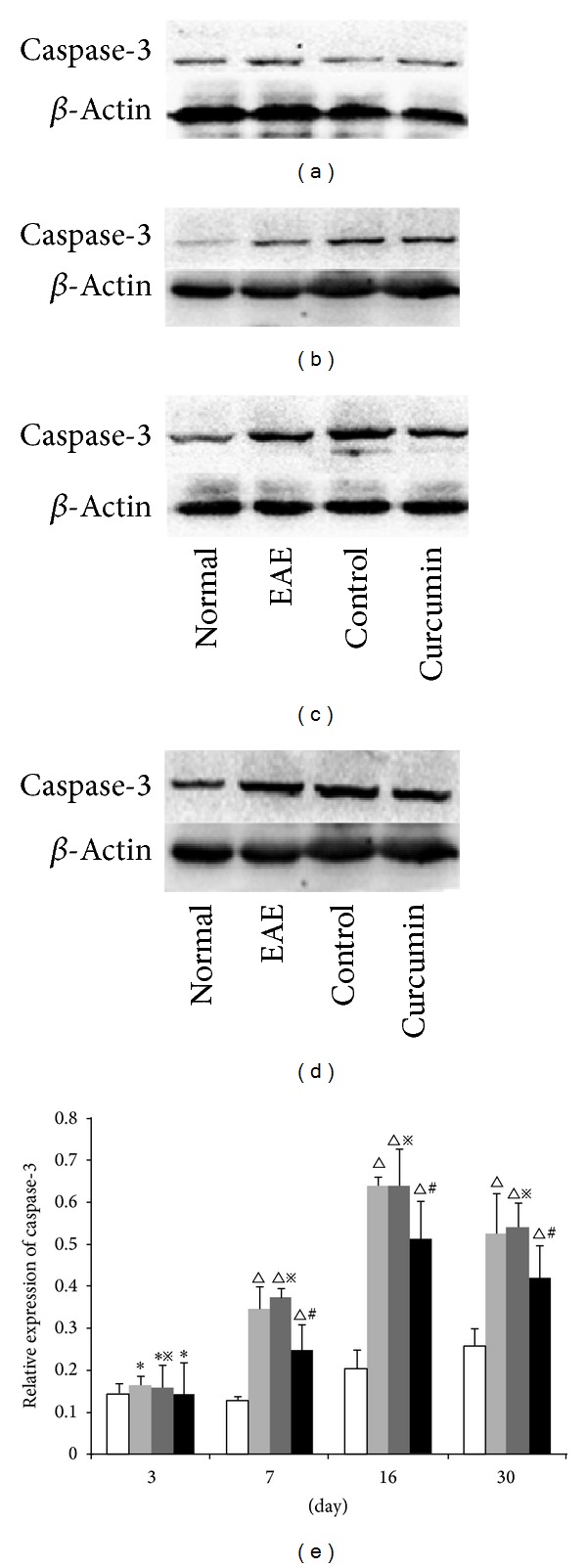
Expression of caspase-3 in each group detected by western blot. (a) 3-day group, (b) 7-day group, (c) 16-day group, and (d) 30-day group. (e) Bars represent relative protein expression of caspase-3 related to *β*-actin. Data are presented as mean ± SD; *P > 0.05, compared to the normal group; ^△^ *P* < 0.01, compared to the normal group; ^#^ *P* < 0.05, compared to the EAE group at the same time point; ^*※*^ *P* > 0.05, compared to the EAE group at the same time point.

The expression of caspase-3, a specific apoptosis marker, was evaluated at different time points using western blot. There was a relatively low expression of caspase-3 in the normal group. The expression levels remained low with no significant differences observed between any of the groups 3 days p.i (*P* > 0.05). In the EAE group, the expression of caspase-3 increased at 7 days p.i. and reached peak levels at 16 days (*P* < 0.01) and then dropped a little at 30 days (*P* < 0.01). The expression of caspase-3 in the curcumin group was significantly lower at the same time points than the EAE group for the 7-day, 16-day, and 30-day groups (*P* < 0.05) (Figure 6).

### 3.7. Cyt-c Expression Levels Detected by Western Blot

**Figure 7:**
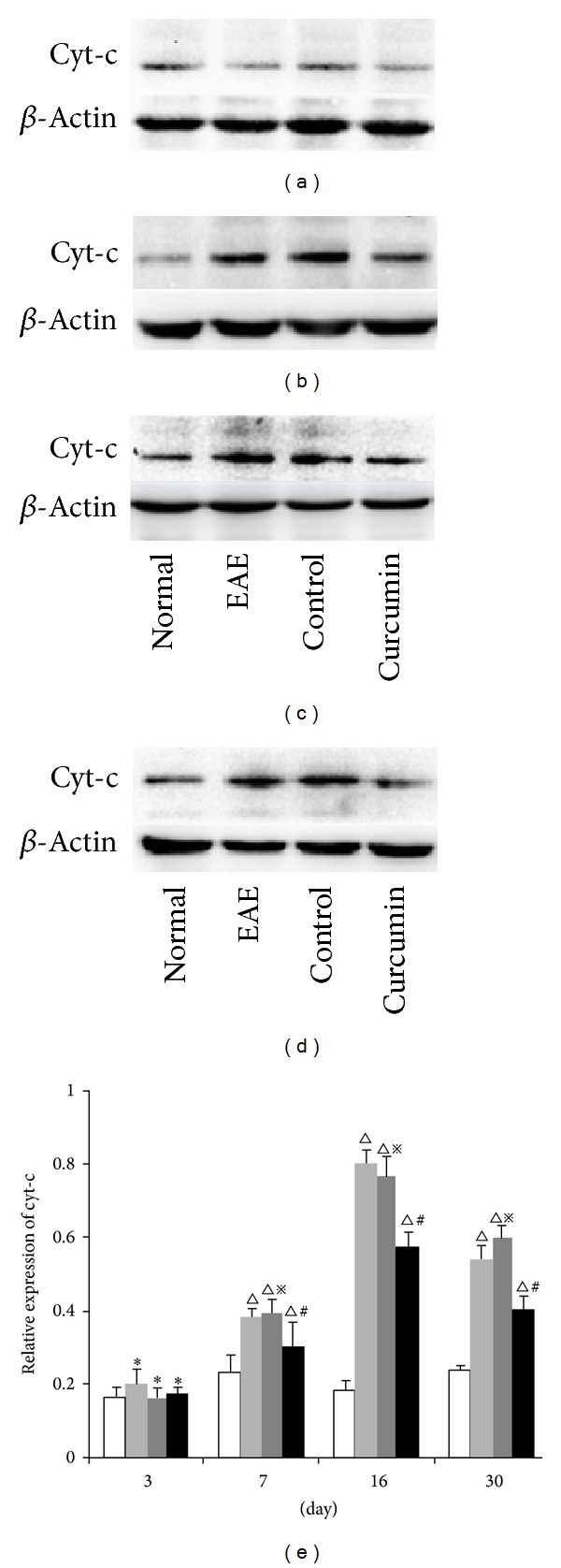
Expression of cyt-c in each group detected by western blot. (a) 3-day group, (b) 7-day group, (c) 16-day group, (d) 30-day group. (e): Bars represent relative protein expression of cyt-c related to *β*-actin. Data are presented as mean ± SD; *P > 0.05, compared to the normal group; ^△^ *P* < 0.01, compared to the normal group; ^#^ *P* < 0.05, compared to the EAE group at the same time point; ^*※*^ *P* > 0.05, compared to the EAE group at the same time point.

The expression tendency of cyt-c was consistent with caspase-9. No significant difference was observed between the 3-day groups and the normal group (*P* > 0.05). The expression of cyt-c increased at 7 days p.i. and reached peak levels at 16 days (*P* < 0.01). The expression at 30 days was lower than at 16 days (*P* < 0.01). The expression in the curcumin group was significantly lower than in the EAE group at the same time points at 7, 16, and 30 days (*P* < 0.05) (Figure 7).

### 3.8. Cleaved-Caspase-9 Expression Levels Detected by Western Blot

**Figure 8:**
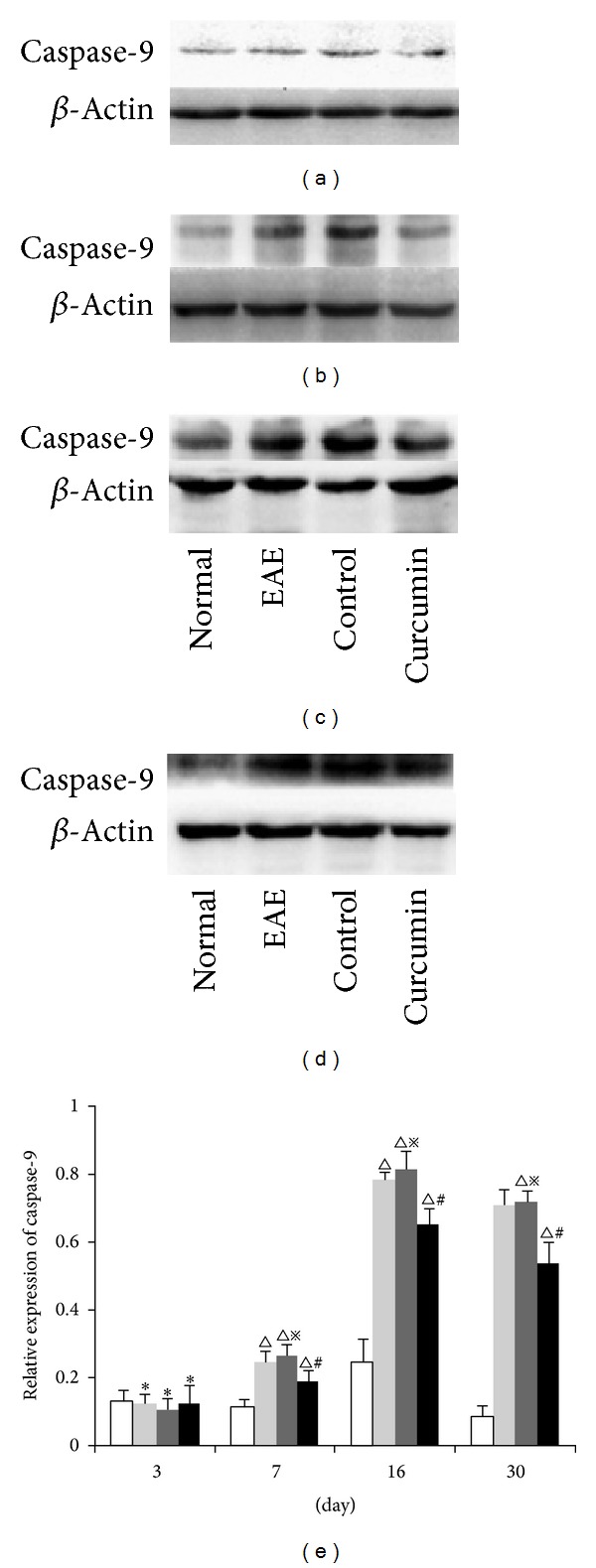
Expression of cleaved caspase-9 in each group detected by western blot. (a) 3-day group, (b) 7-day group, (c) 16-day group, and (d) 30-day group. (e) Bars represent relative protein expression of cleaved caspase-9 related to *β*-actin. Data are presented as mean ± SD; *P > 0.05, compared to the normal group; ^△^ *P* < 0.01, compared to the normal group; ^#^ *P* < 0.05, compared to the EAE group at the same time point; ^*※*^ *P* > 0.05, compared to the EAE group at the same time point.

Caspase-9 is a specific marker for mitochondrial apoptosis. The expression of cleaved caspase-9 was evaluated by western blot. No significant differences were observed between any of the three groups at 3 days p.i. and the normal group (*P* > 0.05). In the EAE group, expression of caspase-9 increased at 7 days (*P* < 0.01) and reached peak levels at 16 days (*P* < 0.01). The expression in the curcumin group was significantly lower than in the EAE group at the same time point by 7, 16, and 30 days (Figure 8).

## 4. Discussion

There is a paucity of reports concerning the pathological characteristics of the whole course post-EAE. In this study, for the first time, we explored apoptosis and mitochondrial injury at different stages (early stage: 3d and 7d; acute stage: 16d; chronic stage: 30d) of EAE. Barnett et al. found that oligodendrocyte apoptosis occurred in the early stages when new lesions were formed in MS [3, 4]. However, whether oligodendrocyte apoptosis occurs in the early stages of EAE is unknown. We found that TUNEL-positive cells occurred as early as 3 days after the EAE model was conducted. The symptomatic severity of EAE was consistent with the number of TUNEL-positive cells. The apoptotic cells in the early stages of EAE were confirmed to be oligodendrocytes and neurons. These apoptotic cells were accompanied by intact myelin sheaths and axons, without the typical morphology of EAE lesions. The results suggest that oligodendrocyte and neuronal apoptosis is one of the earliest pathological events in EAE, occurring in the early stages of EAE/MS pathology.

It has been shown that hypoxia-like tissue injury happens in the early stages of MS, which suggests that mitochondrial injury might be involved in the pathogenesis of MS [17]. Mitochondrial injury mediates the intrinsic apoptosis pathway [18]. However, mitochondrial injury in EAE and MS is still under exploration. We found that mitochondrial injury occurred 3 days after EAE was conducted and continued through the rest of the stages of EAE. The results indicate that mitochondrial injury may be one of the main causes for the apoptosis in the early stage of EAE. Mitochondrial injury may be responsible for the chain reaction of demyelination and inflammation after EAE. Clarifying this pathway would require further study.

Curcumin has been shown to ameliorate EAE. The mechanism by which this is done include but is not limited to the inhibition of IL-17 production [12], modulation of CD4^+^ T cell and toll-like receptors (TLRs) [19], and downregulation of IL-12 [20], IFN-*γ*, IL-12, and IL-23 [8]. However, the mechanism and effects of curcumin on apoptosis are unclear. In this study, we first demonstrated that curcumin inhibits apoptosis in the spinal cord in both the acute stage (16 days) and chronic stage (30 days) of EAE, and then we explored the manner in which this occurred. We demonstrated that curcumin can inhibit the expression of activated caspase-3, caspase-9, and cyt-c starting from 7 days p.i. We also demonstrate that curcumin inhibits the decrease of MBP protein, which inhibits demyelination in EAE.

Activation of caspase-3 is the key event in the course of intrinsic apoptosis [21]. Although apoptosis occurred at 3 days p.i, the expression of activated caspase-3 significantly increased until 7 days p.i. The expression of MBP was significantly decreased after 16 days p.i, which manifested in demyelination not occurring until 16 days p.i. This suggested that neuronal apoptosis occurred earlier in the course of the disease than demyelination.

Mitochondria are responsible for the intrinsic apoptosis pathway [22]. Cytochrome-c (cyt-c) release is an essential step in the intrinsic apoptotic cascade [23, 24]. The release of cyt-c into the cytosol triggers the formation of the apoptosome and the activation of the caspase cascade [25]. Caspase-9, known as the initiator of intrinsic apoptosis, binds to the apoptosome and activates the effector caspases-3, which is considered the major enzyme in a cell's commitment to apoptosis [26]. In this study, the expression of activated cyt-c and caspase-9 was elevated from 7 days after the EAE mice were immunized, and the high expression persisted throughout the entire course of EAE. This phenomenon is consistent with the variation of mitochondrial injury and the severity of EAE we observed. We found that curcumin inhibits the expression of activated cyt-c and caspase-9 from 7 days p.i. and also inhibits mitochondrial injury and reduces the number of apoptotic cells. This result illuminates the way that curcumin suppresses the intrinsic apoptotic pathway—it inhibits the release of cyt-c into the cytosol and then reduces the activation of caspase-9. This may be one of the mechanisms by which curcumin inhibits apoptosis in EAE mice. Whether the exogenous apoptotic pathway is also affected by curcumin needs to be investigated in a further study.

This study demonstrated that mitochondrial injury and oligodendrocyte/neuronal apoptosis occur in the early stages of EAE pathology, which then proceeds to demyelination and axonal injury. We also found that curcumin inhibits the apoptosis of EAE from the early stages of EAE. This inhibitory effect occurs through mitochondrial protection and inhibition of the intrinsic apoptotic pathway.

## Figures and Tables

**Table 1:** Expression of TUNEL (+) cells in the spinal cord ($\overset{-}{x}$ ± *s*, %).

Groups	Normal group	3-day group	7-day group	16-day group	30-day group
TUNEL (+) cells	0.503 ± 0.541	2.472 ± 0.472^△^	2.845 ± 0.517^△∗^	3.964 ± 0.944^△#^	3.325 ± 0.630^△#^

^△^ *P* < 0.01, compared to the normal group; *P > 0.05, compared to the 3-day group; ^#^ *P* < 0.05, compared to the 3-day group.

**Table 2:** Expression of TUNEL (+) cells in the spinal cord of EAE mice p.i. ($\overset{-}{x}\pm s$, %).

Groups	16 days	30 days
EAE group	3.968 ± 0.94	3.02 ± 0.634^▲^
Control group	3.846 ± 0.682^#^	2.951 ± 0.422^#^
Curcumin group	2.361 ± 0.211^△^	1.756 ± 0.964^△▲^

^#^ *P* > 0.05, compared to the EAE group at the same time point; ^△^ *P* < 0.01, compared to the EAE group at the same time point; ^▲^ *P* < 0.05, compared to 16 days in the same group.

## Abbreviations

BCG: Bacillus calmette-guerin
CFA: Complete Freund's adjuvant
EAE: Experimental autoimmune encephalomyelitis
p.i.: Postimmunization
MS: Multiple sclerosis.
